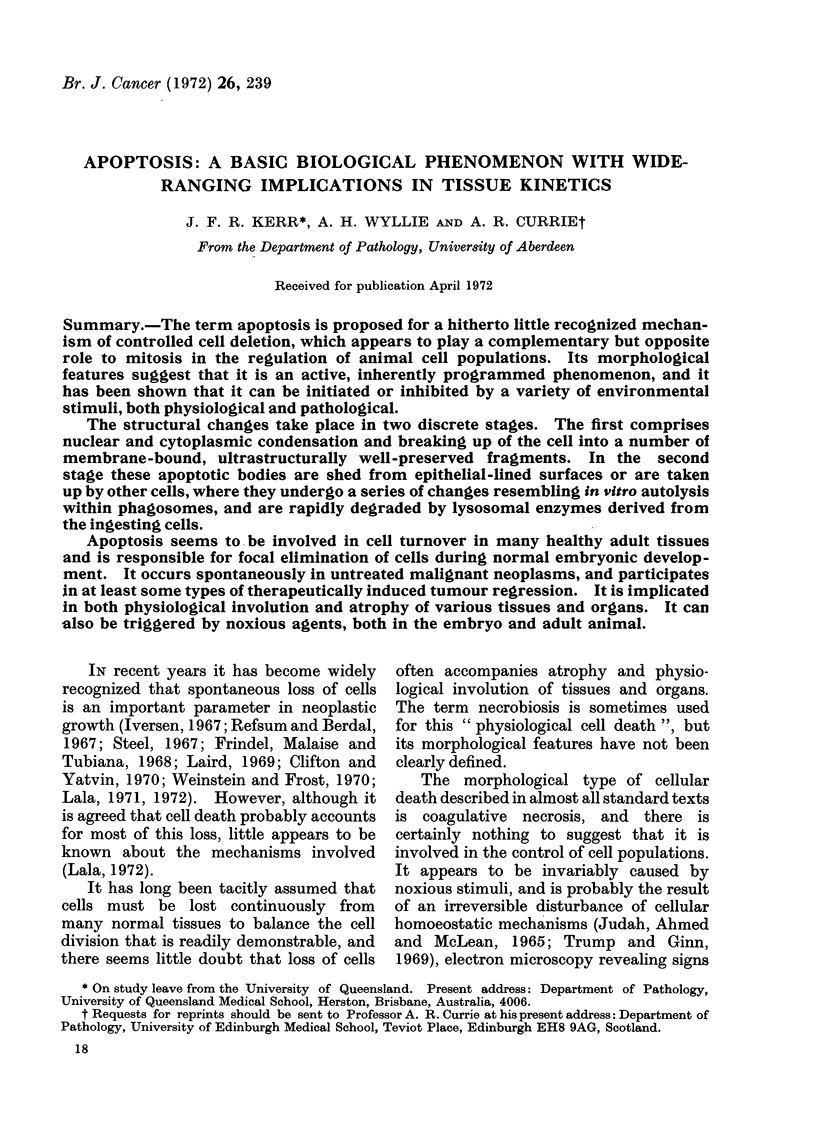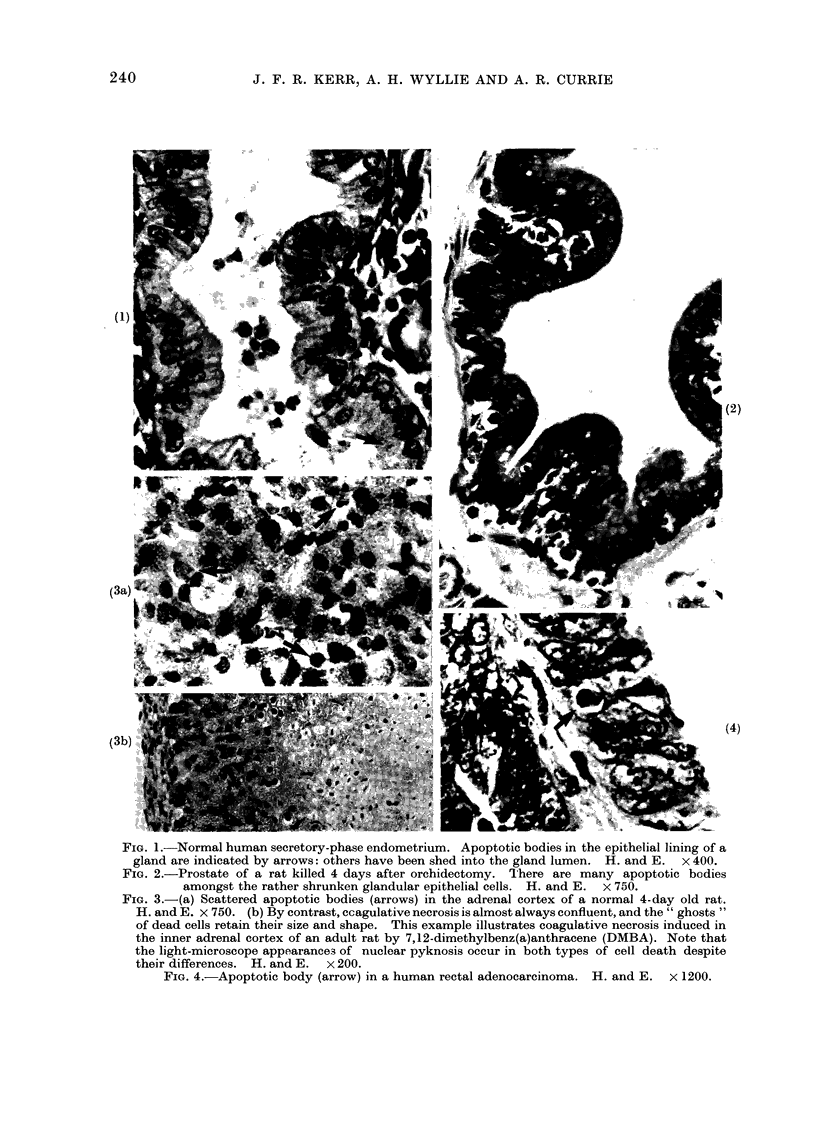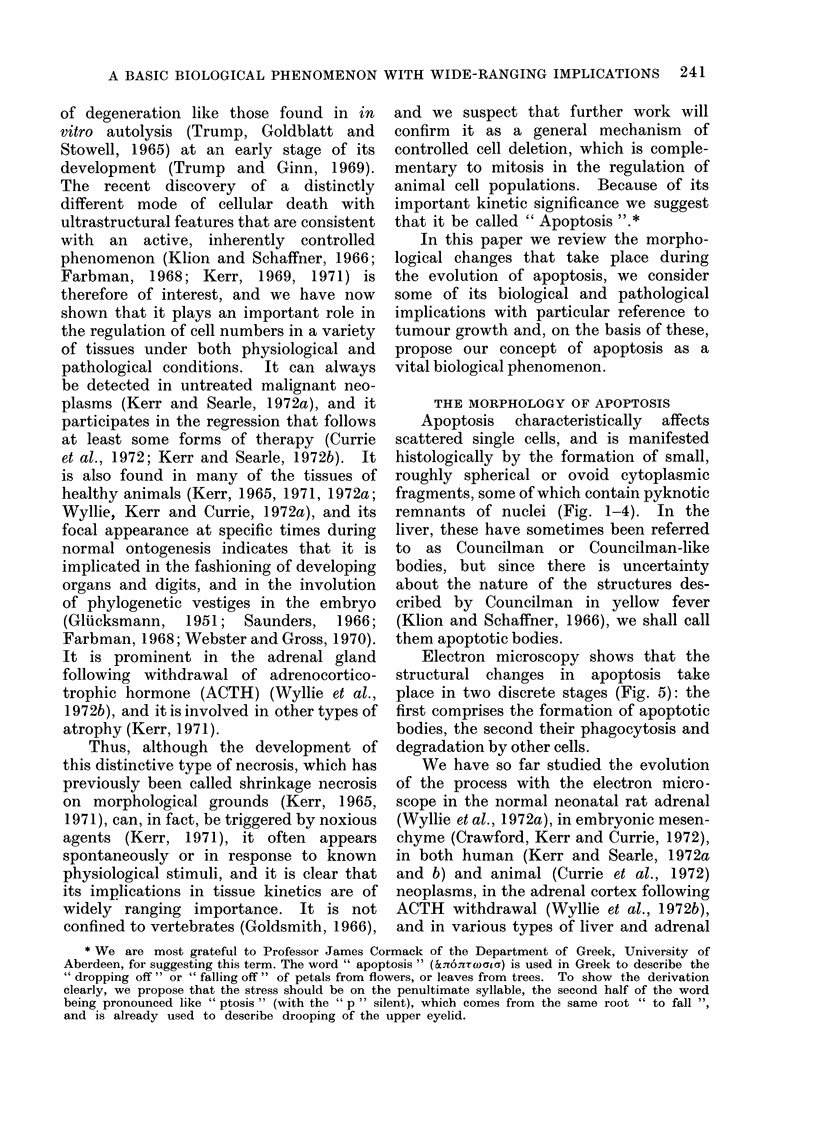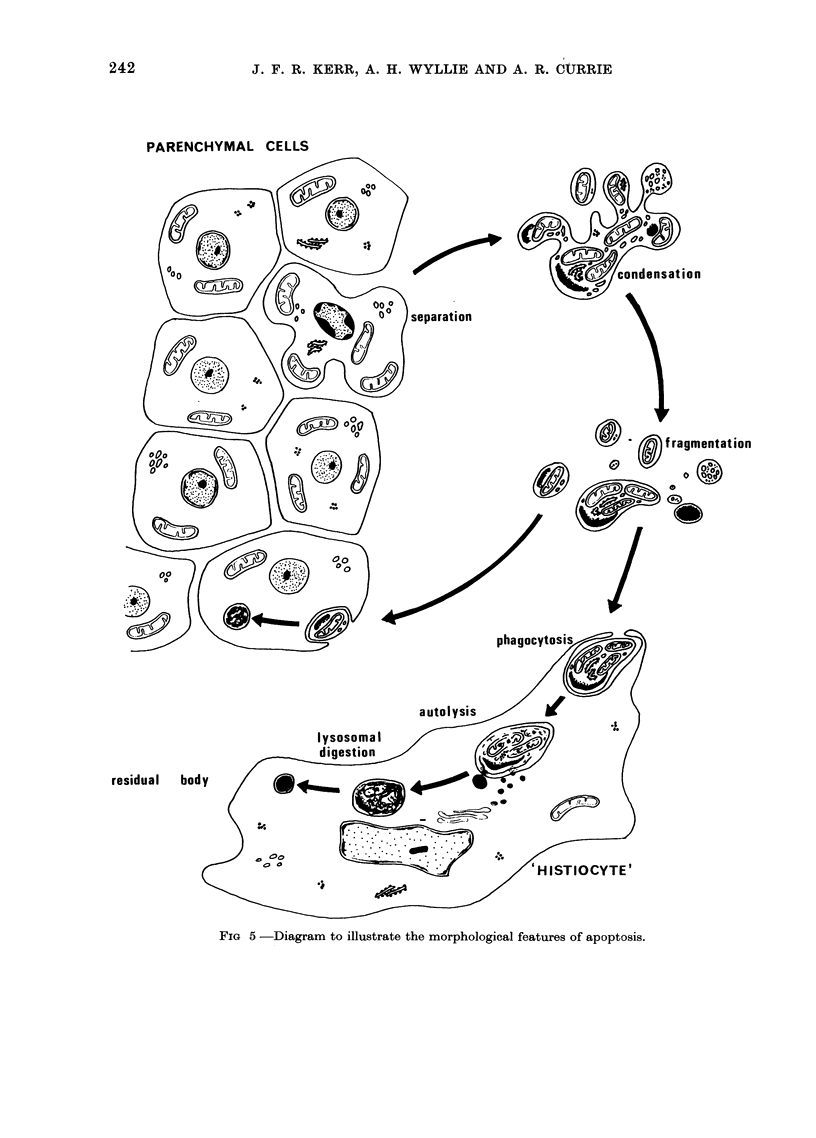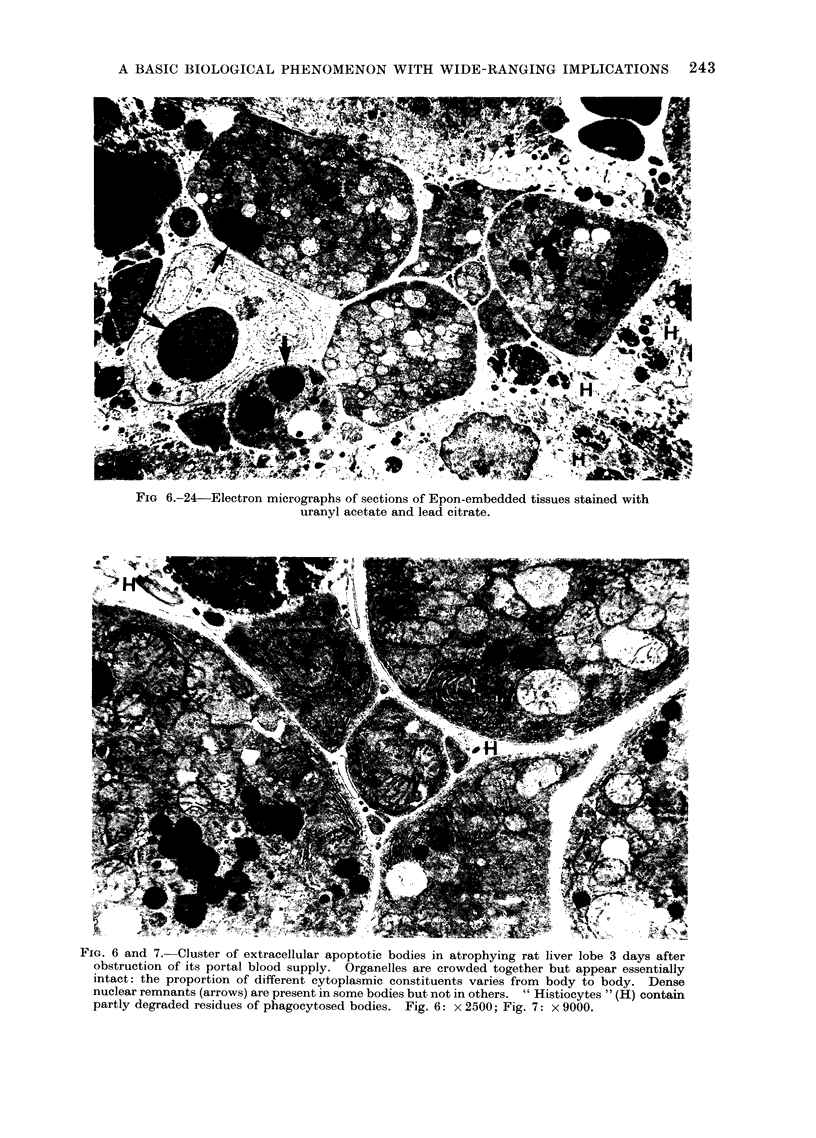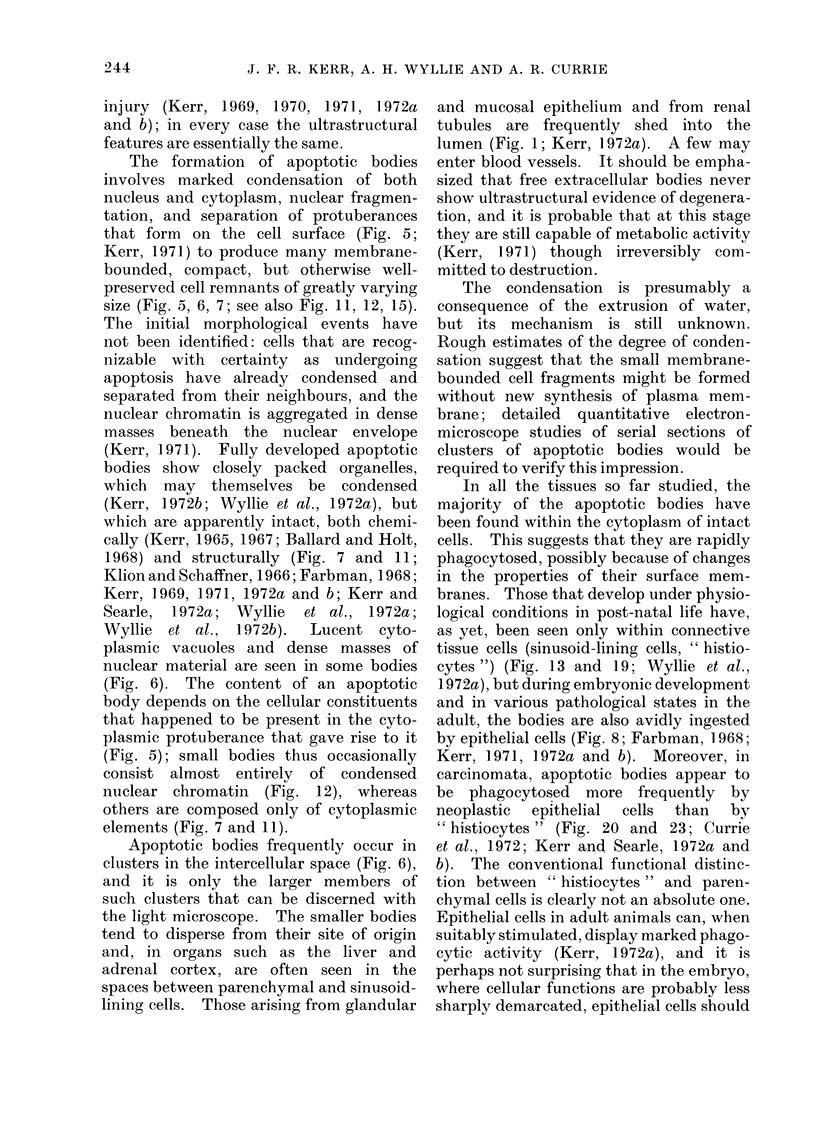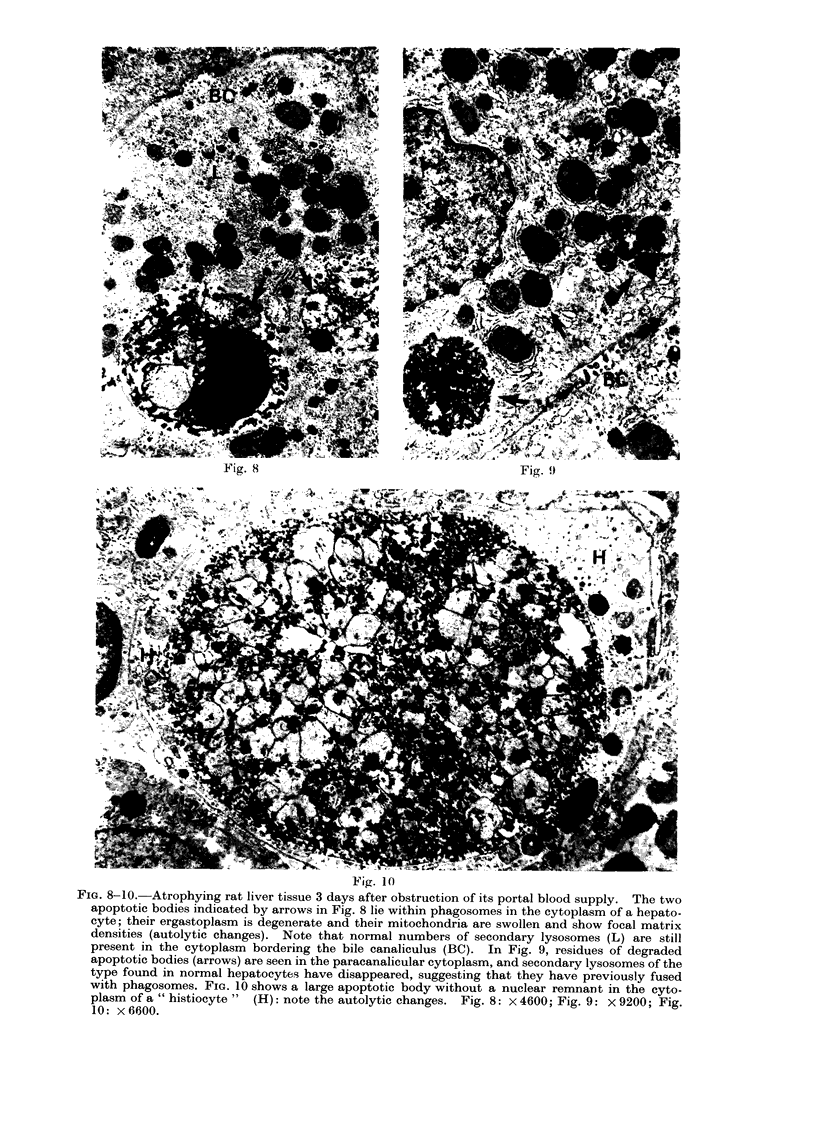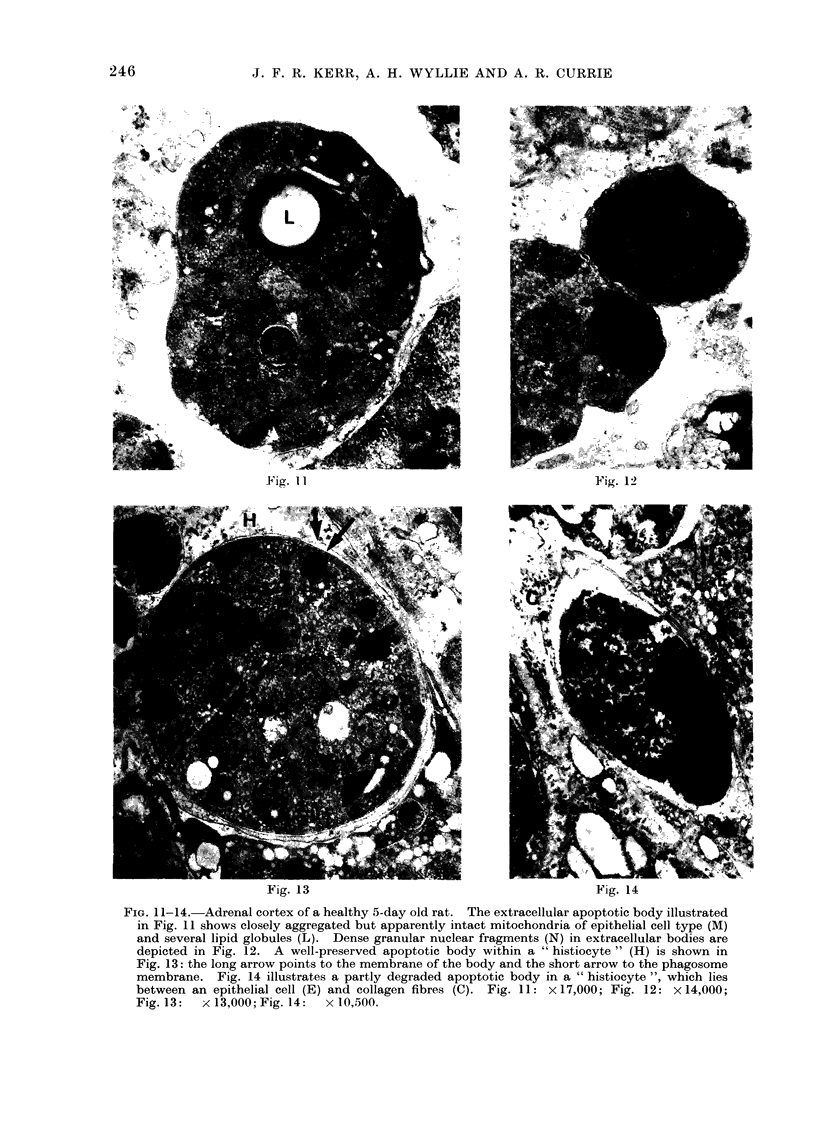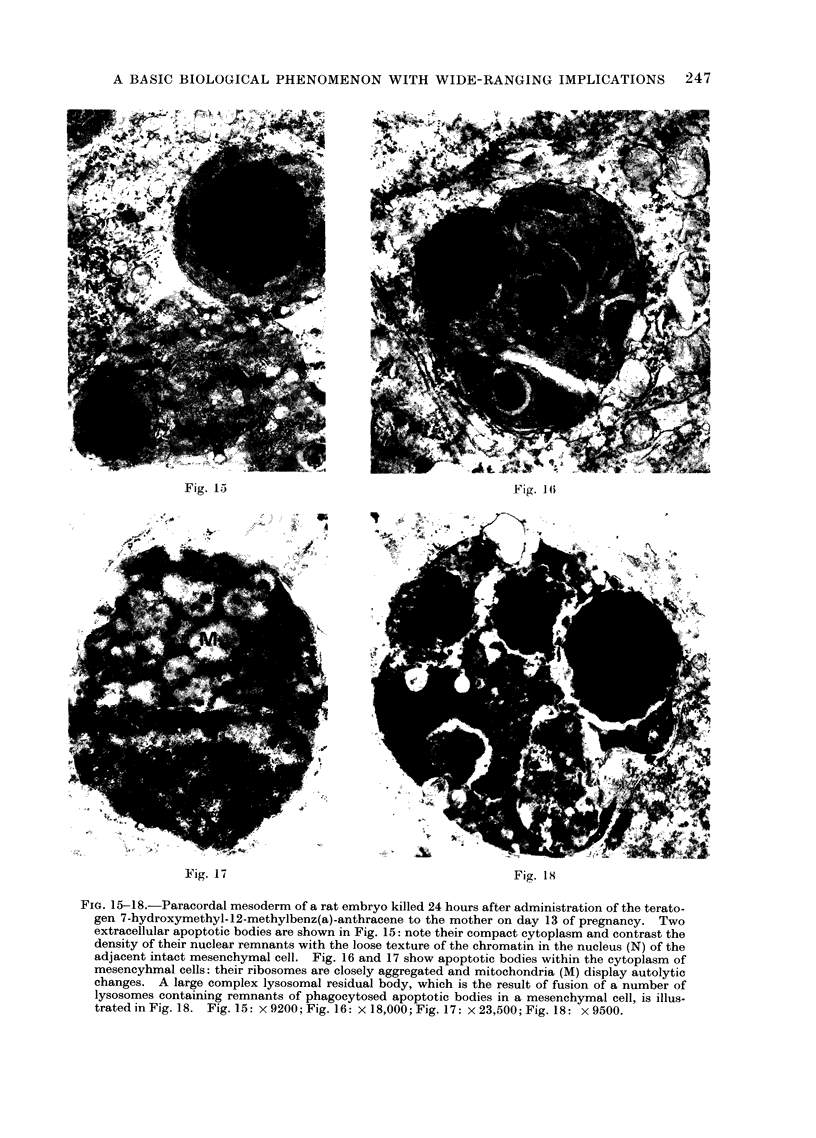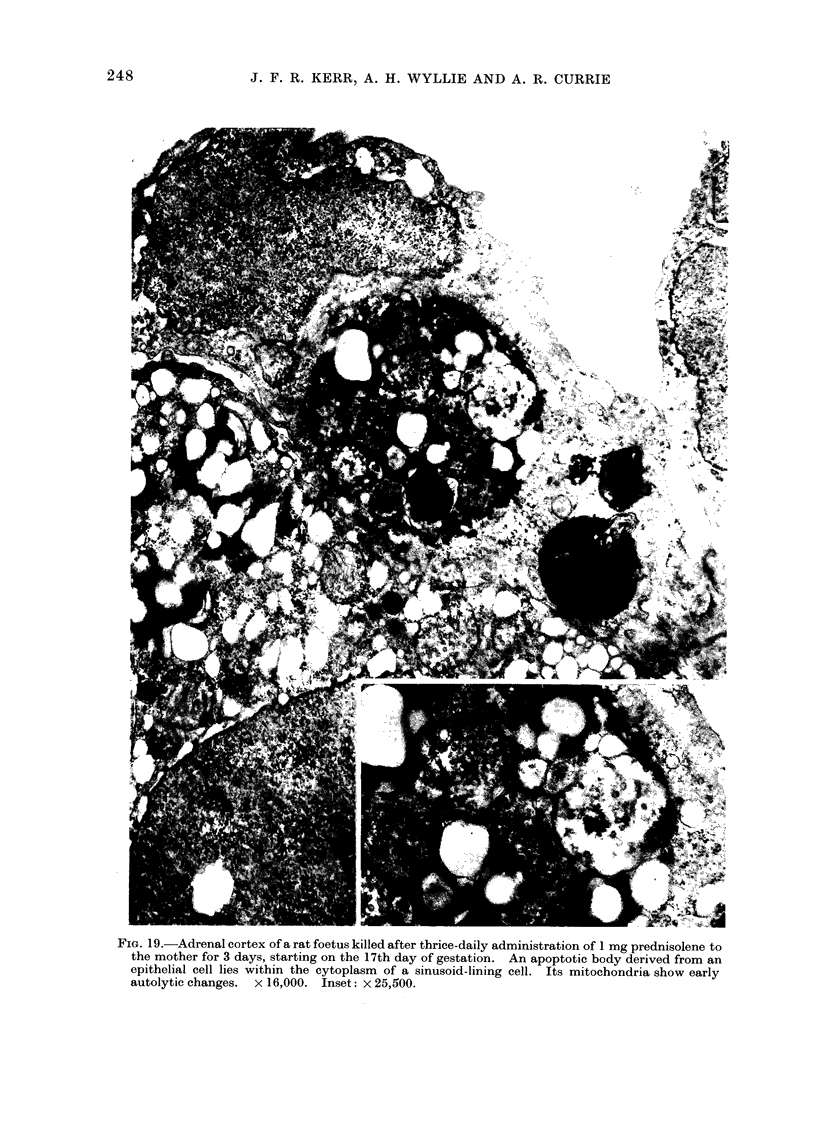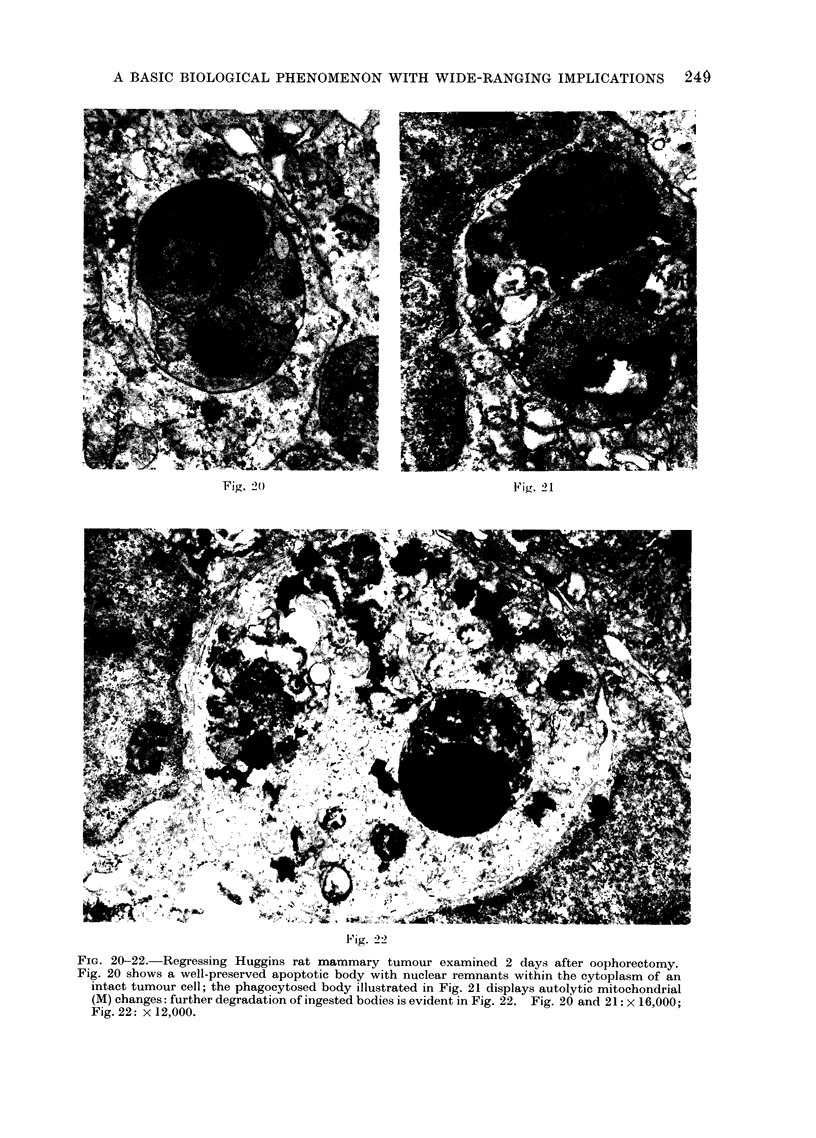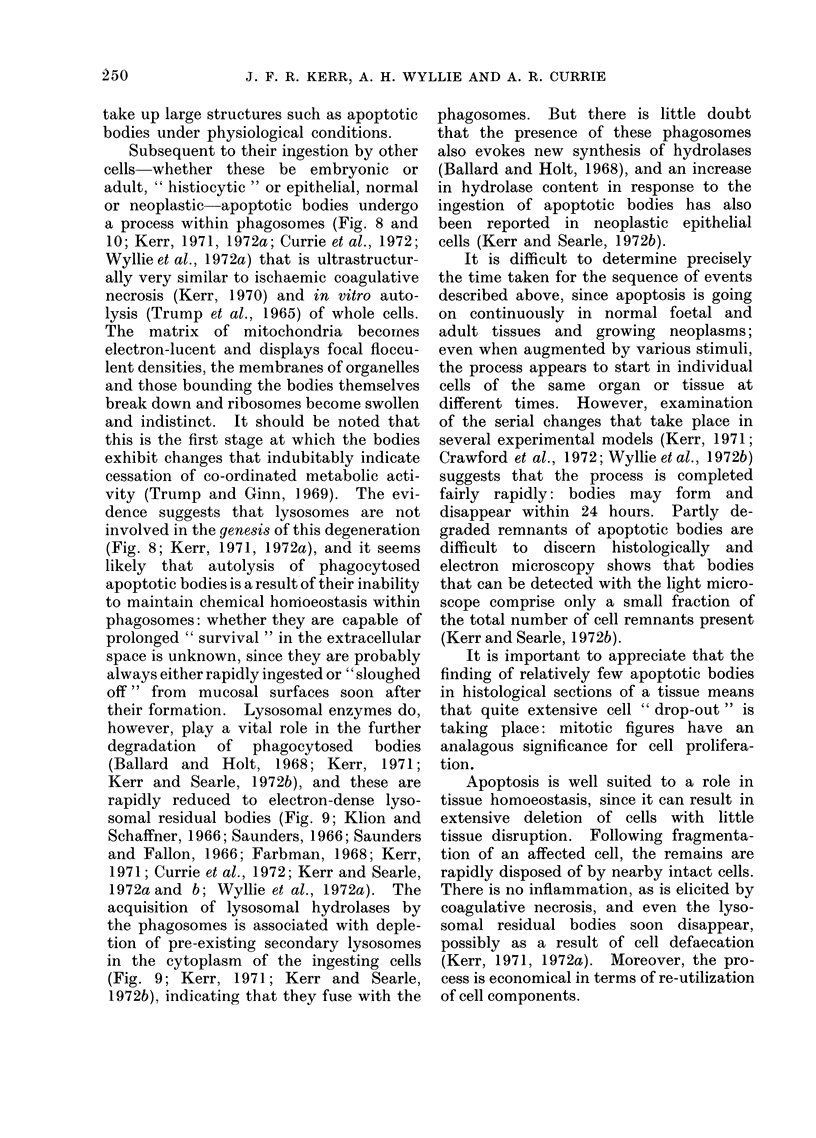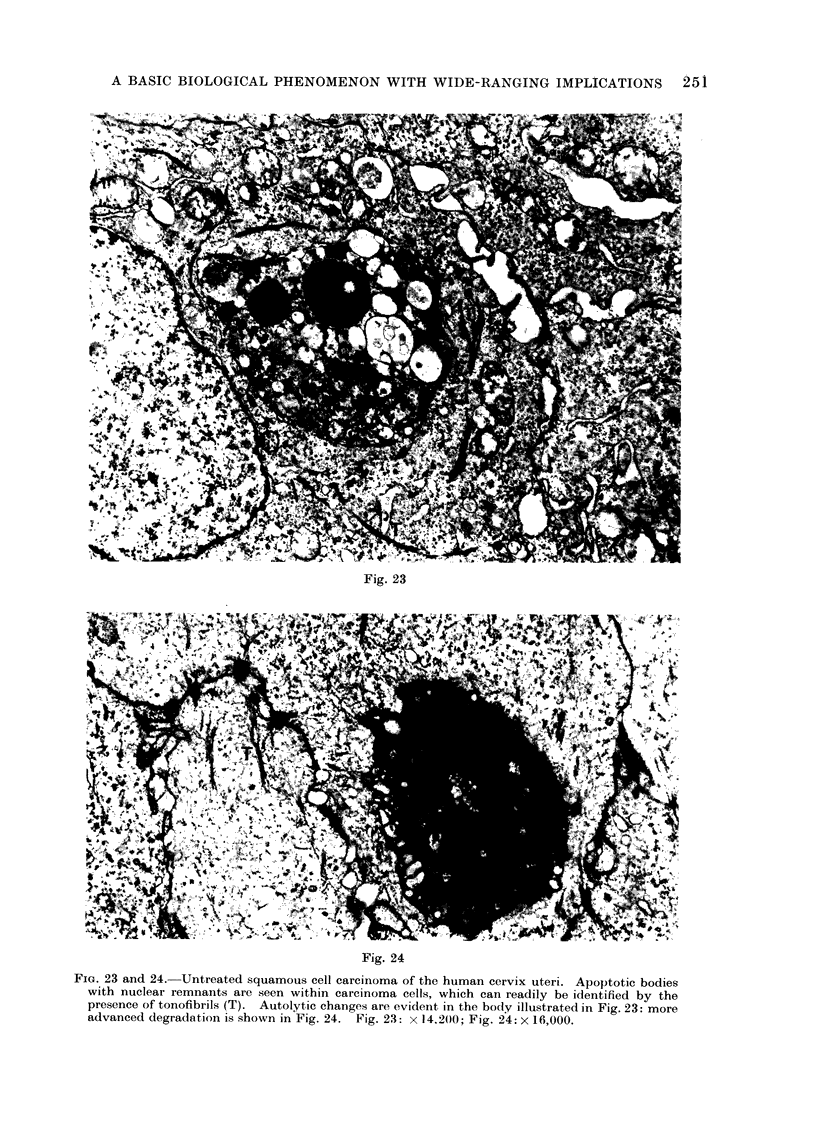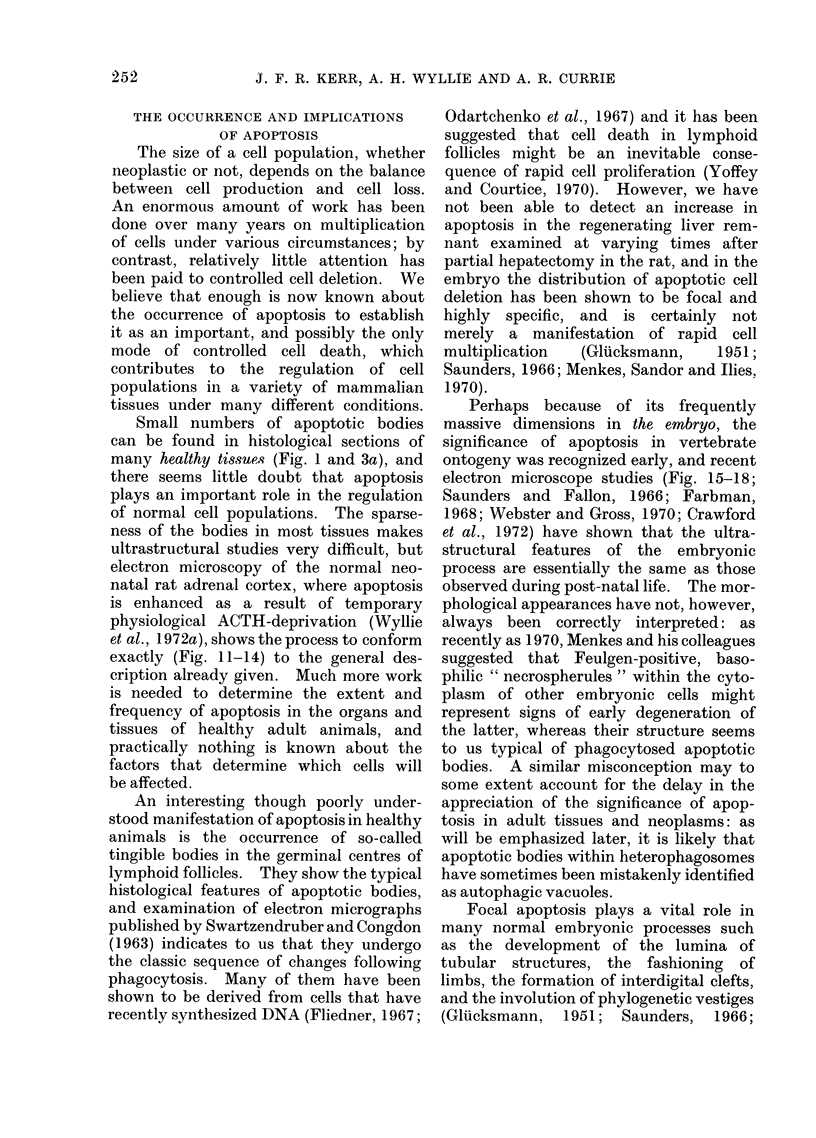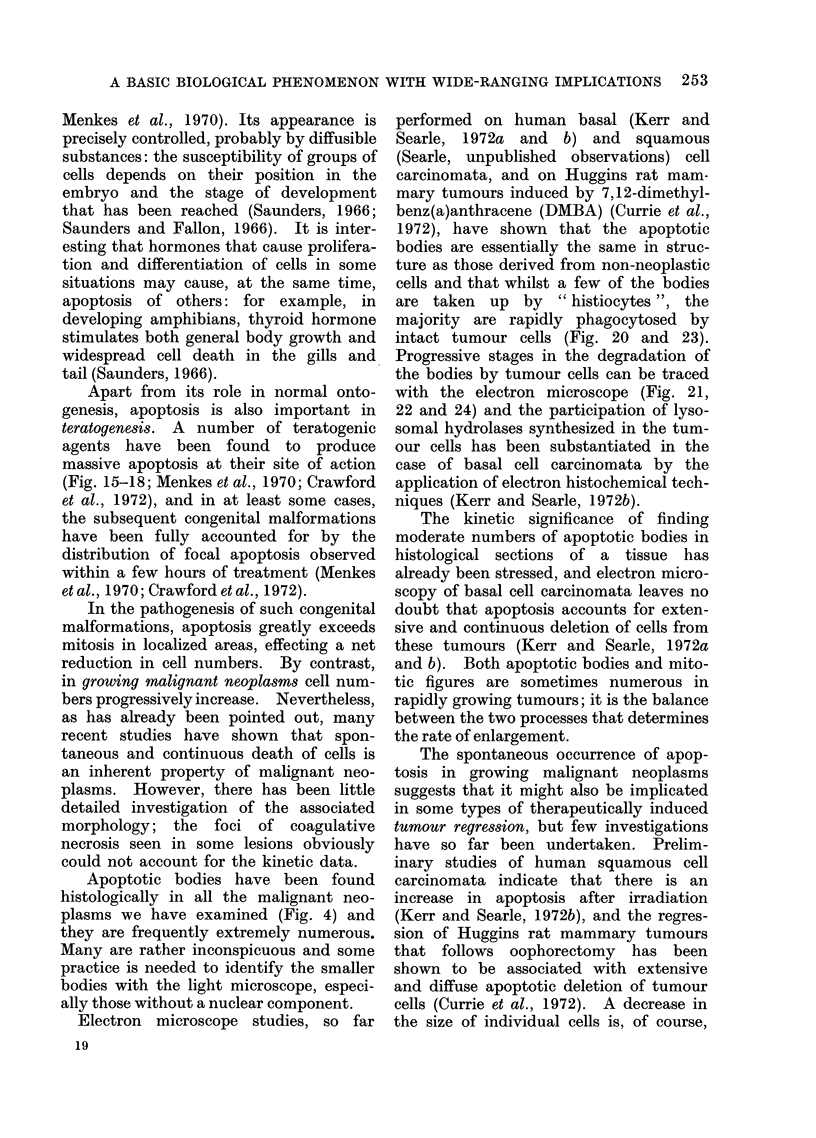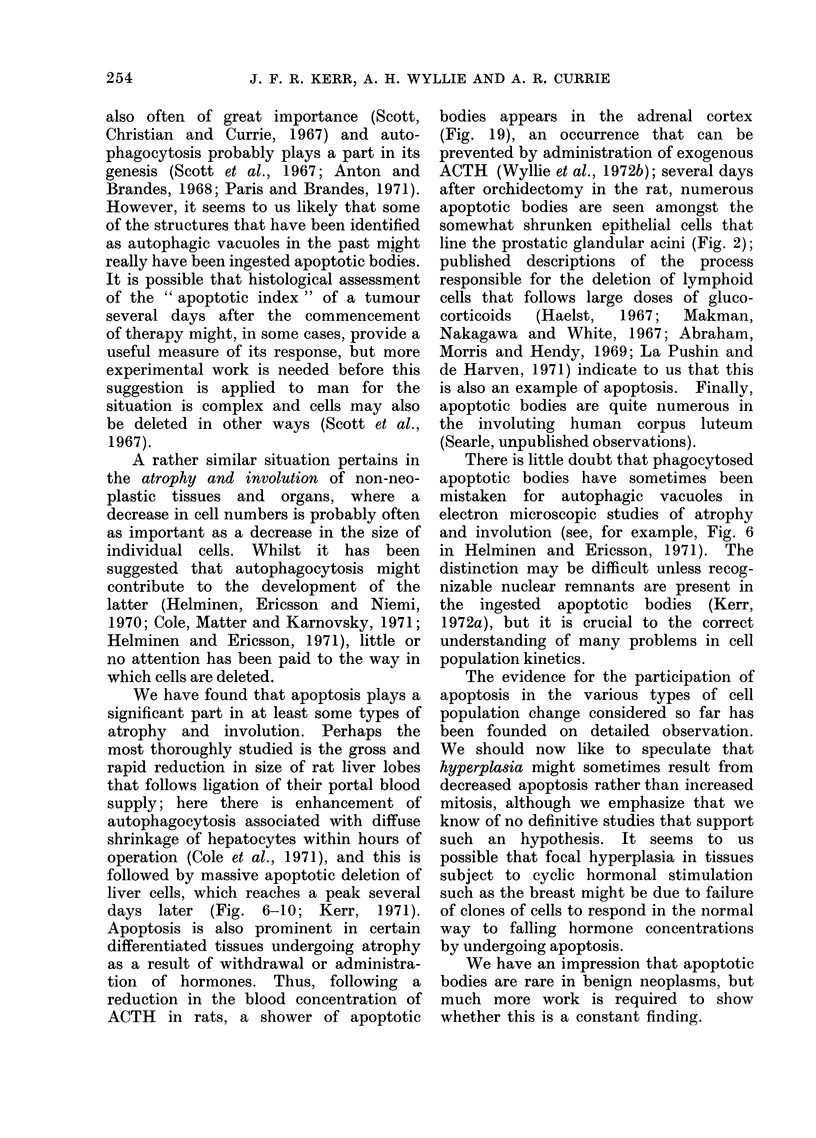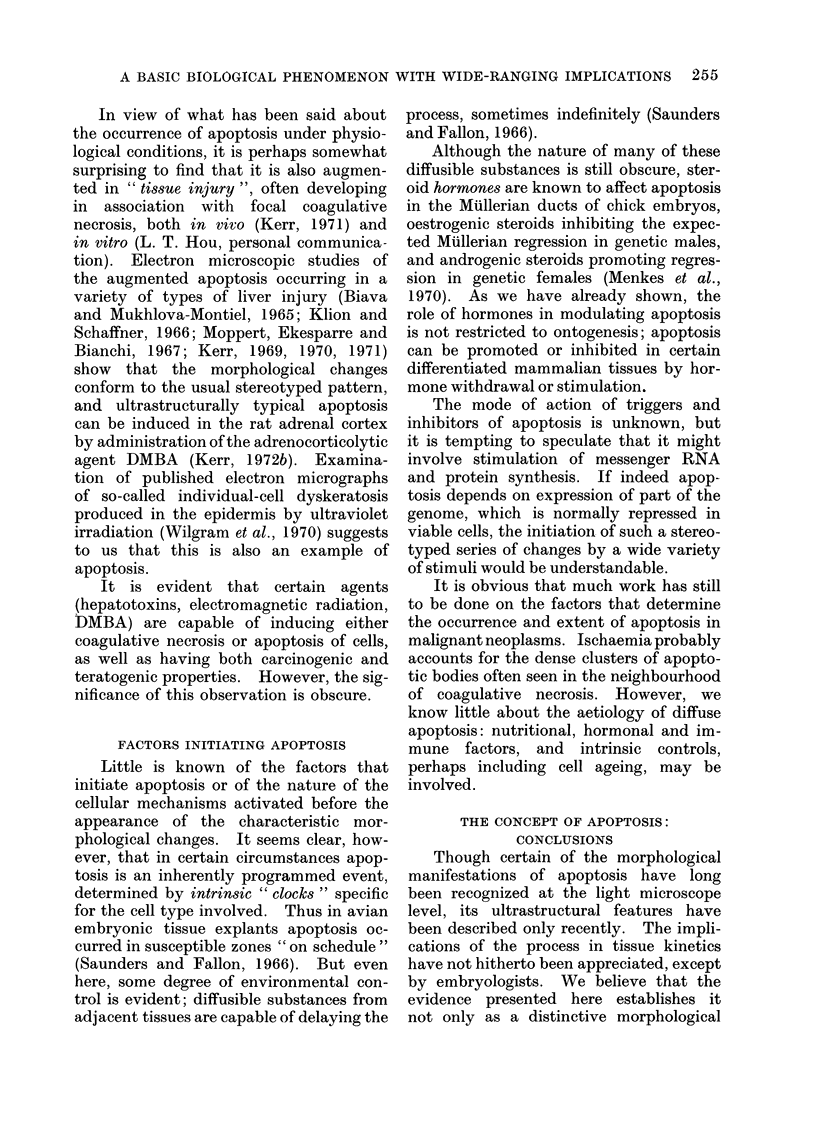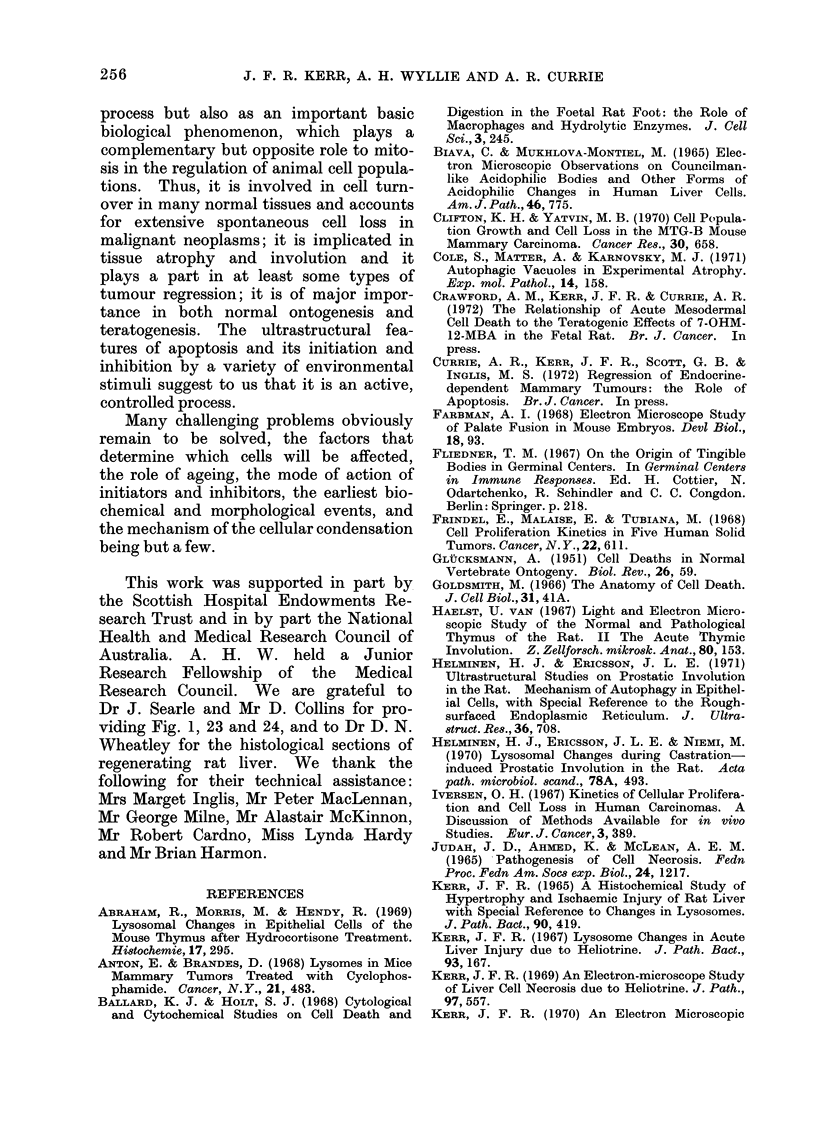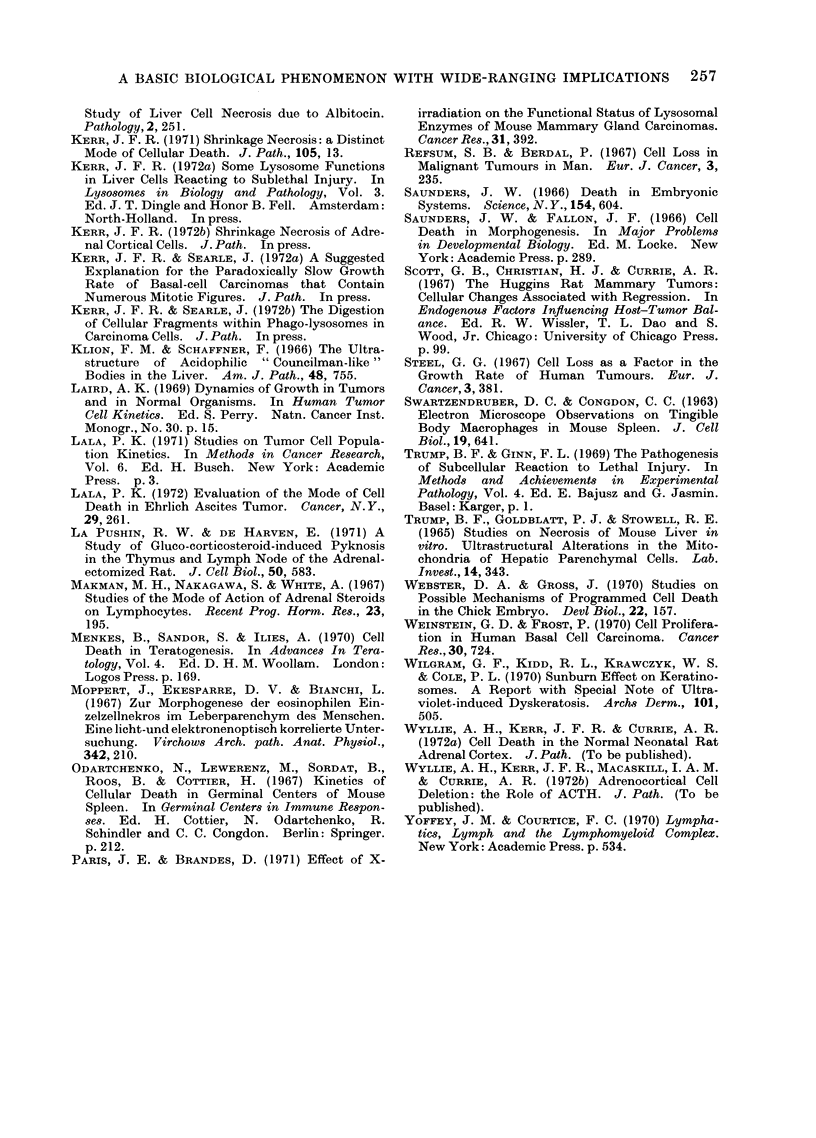# Apoptosis: A Basic Biological Phenomenon with Wide-ranging Implications in Tissue Kinetics

**DOI:** 10.1038/bjc.1972.33

**Published:** 1972-08

**Authors:** J. F. R. Kerr, A. H. Wyllie, A. R. Currie

## Abstract

**Images:**


*Br. J. Cancer* (1972) **26**, 239

### APOPTOSIS: A BASIC BIOLOGICAL PHENOMENON WITH WIDE-RANGING IMPLICATIONS IN TISSUE KINETICS

J. F. R. KERR*, A. H. WYLLIE AND A. R. CURRIE†

*From the Department of Pathology, University of Aberdeen*

Received for publication April 1972

**Summary.—The term apoptosis is proposed for a hitherto little recognized mechanism of controlled cell deletion, which appears to play a complementary but opposite role to mitosis in the regulation of animal cell populations. Its morphological features suggest that it is an active, inherently programmed phenomenon, and it has been shown that it can be initiated or inhibited by a variety of environmental stimuli, both physiological and pathological.**

**The structural changes take place in two discrete stages. The first comprises nuclear and cytoplasmic condensation and breaking up of the cell into a number of membrane-bound, ultrastructurally well-preserved fragments. In the second stage these apoptotic bodies are shed from epithelial-lined surfaces or are taken up by other cells, where they undergo a series of changes resembling *in vitro* autolysis within phagosomes, and are rapidly degraded by lysosomal enzymes derived from the ingesting cells.**

**Apoptosis seems to be involved in cell turnover in many healthy adult tissues and is responsible for focal elimination of cells during normal embryonic development. It occurs spontaneously in untreated malignant neoplasms, and participates in at least some types of therapeutically induced tumour regression. It is implicated in both physiological involution and atrophy of various tissues and organs. It can also be triggered by noxious agents, both in the embryo and adult animal.**

IN recent years it has become widely recognized that spontaneous loss of cells is an important parameter in neoplastic growth (Iversen, 1967; Refsum and Berdal, 1967; Steel, 1967; Frindel, Malaise and Tubiana, 1968; Laird, 1969; Clifton and Yatvin, 1970; Weinstein and Frost, 1970; Lala, 1971, 1972). However, although it is agreed that cell death probably accounts for most of this loss, little appears to be known about the mechanisms involved (Lala, 1972).

It has long been tacitly assumed that cells must be lost continuously from many normal tissues to balance the cell division that is readily demonstrable, and there seems little doubt that loss of cells often accompanies atrophy and physiological involution of tissues and organs. The term necrobiosis is sometimes used for this " physiological cell death ", but its morphological features have not been clearly defined.

The morphological type of cellular death described in almost all standard texts is coagulative necrosis, and there is certainly nothing to suggest that it is involved in the control of cell populations. It appears to be invariably caused by noxious stimuli, and is probably the result of an irreversible disturbance of cellular homoeostatic mechanisms (Judah, Ahmed and McLean, 1965; Trump and Ginn, 1969), electron microscopy revealing signs

* On study leave from the University of Queensland. Present address: Department of Pathology, University of Queensland Medical School, Herston, Brisbane, Australia, 4006.

† Requests for reprints should be sent to Professor A. R. Currie at his present address: Department of Pathology, University of Edinburgh Medical School, Teviot Place, Edinburgh EH8 9AG, Scotland.

FIG. 1.—Normal human secretory-phase endometrium. Apoptotic bodies in the epithelial lining of a gland are indicated by arrows: others have been shed into the gland lumen. H. and E. ×400.

FIG. 2.—Prostate of a rat killed 4 days after orchidectomy. There are many apoptotic bodies amongst the rather shrunken glandular epithelial cells. H. and E. ×750.

FIG. 3.—(a) Scattered apoptotic bodies (arrows) in the adrenal cortex of a normal 4-day old rat. H. and E. ×750. (b) By contrast, coagulative necrosis is almost always confluent, and the " ghosts " of dead cells retain their size and shape. This example illustrates coagulative necrosis induced in the inner adrenal cortex of an adult rat by 7,12-dimethylbenz(a)anthracene (DMBA). Note that the light-microscope appearances of nuclear pyknosis occur in both types of cell death despite their differences. H. and E. ×200.

FIG. 4.—Apoptotic body (arrow) in a human rectal adenocarcinoma. H. and E. ×1200.

of degeneration like those found in *in vitro* autolysis (Trump, Goldblatt and Stowell, 1965) at an early stage of its development (Trump and Ginn, 1969). The recent discovery of a distinctly different mode of cellular death with ultrastructural features that are consistent with an active, inherently controlled phenomenon (Klion and Schaffner, 1966; Farbman, 1968; Kerr, 1969, 1971) is therefore of interest, and we have now shown that it plays an important role in the regulation of cell numbers in a variety of tissues under both physiological and pathological conditions. It can always be detected in untreated malignant neoplasms (Kerr and Searle, 1972*a*), and it participates in the regression that follows at least some forms of therapy (Currie *et al.*, 1972; Kerr and Searle, 1972*b*). It is also found in many of the tissues of healthy animals (Kerr, 1965, 1971, 1972*a*; Wyllie, Kerr and Currie, 1972*a*), and its focal appearance at specific times during normal ontogenesis indicates that it is implicated in the fashioning of developing organs and digits, and in the involution of phylogenetic vestiges in the embryo (Glücksmann, 1951; Saunders, 1966; Farbman, 1968; Webster and Gross, 1970). It is prominent in the adrenal gland following withdrawal of adrenocorticotrophic hormone (ACTH) (Wyllie *et al.*, 1972*b*), and it is involved in other types of atrophy (Kerr, 1971).

Thus, although the development of this distinctive type of necrosis, which has previously been called shrinkage necrosis on morphological grounds (Kerr, 1965, 1971), can, in fact, be triggered by noxious agents (Kerr, 1971), it often appears spontaneously or in response to known physiological stimuli, and it is clear that its implications in tissue kinetics are of widely ranging importance. It is not confined to vertebrates (Goldsmith, 1966), and we suspect that further work will confirm it as a general mechanism of controlled cell deletion, which is complementary to mitosis in the regulation of animal cell populations. Because of its important kinetic significance we suggest that it be called " Apoptosis ".*

In this paper we review the morphological changes that take place during the evolution of apoptosis, we consider some of its biological and pathological implications with particular reference to tumour growth and, on the basis of these, propose our concept of apoptosis as a vital biological phenomenon.

### THE MORPHOLOGY OF APOPTOSIS

Apoptosis characteristically affects scattered single cells, and is manifested histologically by the formation of small, roughly spherical or ovoid cytoplasmic fragments, some of which contain pyknotic remnants of nuclei (Fig. 1–4). In the liver, these have sometimes been referred to as Councilman or Councilman-like bodies, but since there is uncertainty about the nature of the structures described by Councilman in yellow fever (Klion and Schaffner, 1966), we shall call them apoptotic bodies.

Electron microscopy shows that the structural changes in apoptosis take place in two discrete stages (Fig. 5): the first comprises the formation of apoptotic bodies, the second their phagocytosis and degradation by other cells.

We have so far studied the evolution of the process with the electron microscope in the normal neonatal rat adrenal (Wyllie *et al.*, 1972*a*), in embryonic mesenchyme (Crawford, Kerr and Currie, 1972), in both human (Kerr and Searle, 1972*a* and *b*) and animal (Currie *et al.*, 1972) neoplasms, in the adrenal cortex following ACTH withdrawal (Wyllie *et al.*, 1972*b*), and in various types of liver and adrenal

* We are most grateful to Professor James Cormack of the Department of Greek, University of Aberdeen, for suggesting this term. The word " apoptosis " (ἀπόπτωσισ) is used in Greek to describe the " dropping off " or " falling off " of petals from flowers, or leaves from trees. To show the derivation clearly, we propose that the stress should be on the penultimate syllable, the second half of the word being pronounced like " ptosis " (with the " p " silent), which comes from the same root " to fall ", and is already used to describe drooping of the upper eyelid.

FIG 5 —Diagram to illustrate the morphological features of apoptosis.

FIG 6.–24—Electron micrographs of sections of Epon-embedded tissues stained with uranyl acetate and lead citrate.

FIG. 6 and 7.—Cluster of extracellular apoptotic bodies in atrophying rat liver lobe 3 days after obstruction of its portal blood supply. Organelles are crowded together but appear essentially intact: the proportion of different cytoplasmic constituents varies from body to body. Dense nuclear remnants (arrows) are present in some bodies but not in others. " Histiocytes " (H) contain partly degraded residues of phagocytosed bodies. Fig. 6: ×2500; Fig. 7: ×9000.

injury (Kerr, 1969, 1970, 1971, 1972*a* and *b*); in every case the ultrastructural features are essentially the same.

The formation of apoptotic bodies involves marked condensation of both nucleus and cytoplasm, nuclear fragmentation, and separation of protuberances that form on the cell surface (Fig. 5; Kerr, 1971) to produce many membrane-bounded, compact, but otherwise well-preserved cell remnants of greatly varying size (Fig. 5, 6, 7; see also Fig. 11, 12, 15). The initial morphological events have not been identified: cells that are recognizable with certainty as undergoing apoptosis have already condensed and separated from their neighbours, and the nuclear chromatin is aggregated in dense masses beneath the nuclear envelope (Kerr, 1971). Fully developed apoptotic bodies show closely packed organelles, which may themselves be condensed (Kerr, 1972*b*; Wyllie *et al.*, 1972*a*), but which are apparently intact, both chemically (Kerr, 1965, 1967; Ballard and Holt, 1968) and structurally (Fig. 7 and 11; Klion and Schaffner, 1966; Farbman, 1968; Kerr, 1969, 1971, 1972*a* and *b*; Kerr and Searle, 1972*a*; Wyllie *et al.*, 1972*a*; Wyllie *et al.*, 1972*b*). Lucent cytoplasmic vacuoles and dense masses of nuclear material are seen in some bodies (Fig. 6). The content of an apoptotic body depends on the cellular constituents that happened to be present in the cytoplasmic protuberance that gave rise to it (Fig. 5); small bodies thus occasionally consist almost entirely of condensed nuclear chromatin (Fig. 12), whereas others are composed only of cytoplasmic elements (Fig. 7 and 11).

Apoptotic bodies frequently occur in clusters in the intercellular space (Fig. 6), and it is only the larger members of such clusters that can be discerned with the light microscope. The smaller bodies tend to disperse from their site of origin and, in organs such as the liver and adrenal cortex, are often seen in the spaces between parenchymal and sinusoid-lining cells. Those arising from glandular and mucosal epithelium and from renal tubules are frequently shed into the lumen (Fig. 1; Kerr, 1972*a*). A few may enter blood vessels. It should be emphasized that free extracellular bodies never show ultrastructural evidence of degeneration, and it is probable that at this stage they are still capable of metabolic activity (Kerr, 1971) though irreversibly committed to destruction.

The condensation is presumably a consequence of the extrusion of water, but its mechanism is still unknown. Rough estimates of the degree of condensation suggest that the small membrane-bounded cell fragments might be formed without new synthesis of plasma membrane; detailed quantitative electron-microscope studies of serial sections of clusters of apoptotic bodies would be required to verify this impression.

In all the tissues so far studied, the majority of the apoptotic bodies have been found within the cytoplasm of intact cells. This suggests that they are rapidly phagocytosed, possibly because of changes in the properties of their surface membranes. Those that develop under physiological conditions in post-natal life have, as yet, been seen only within connective tissue cells (sinusoid-lining cells, " histiocytes ") (Fig. 13 and 19; Wyllie *et al.*, 1972*a*), but during embryonic development and in various pathological states in the adult, the bodies are also avidly ingested by epithelial cells (Fig. 8; Farbman, 1968; Kerr, 1971, 1972*a* and *b*). Moreover, in carcinomata, apoptotic bodies appear to be phagocytosed more frequently by neoplastic epithelial cells than by " histiocytes " (Fig. 20 and 23; Currie *et al.*, 1972; Kerr and Searle, 1972*a* and *b*). The conventional functional distinction between " histiocytes " and parenchymal cells is clearly not an absolute one. Epithelial cells in adult animals can, when suitably stimulated, display marked phagocytic activity (Kerr, 1972*a*), and it is perhaps not surprising that in the embryo, where cellular functions are probably less sharply demarcated, epithelial cells should

Fig. 8

Fig. 9

Fig. 10

FIG. 8–10.—Atrophying rat liver tissue 3 days after obstruction of its portal blood supply. The two apoptotic bodies indicated by arrows in Fig. 8 lie within phagosomes in the cytoplasm of a hepatocyte; their ergastoplasm is degenerate and their mitochondria are swollen and show focal matrix densities (autolytic changes). Note that normal numbers of secondary lysosomes (L) are still present in the cytoplasm bordering the bile canaliculus (BC). In Fig. 9, residues of degraded apoptotic bodies (arrows) are seen in the paracanalicular cytoplasm, and secondary lysosomes of the type found in normal hepatocytes have disappeared, suggesting that they have previously fused with phagosomes. FIG. 10 shows a large apoptotic body without a nuclear remnant in the cytoplasm of a " histiocyte " (H): note the autolytic changes. Fig. 8: ×4600; Fig. 9: ×9200; Fig. 10: ×6600.

Fig. 11

Fig. 12

Fig. 13

Fig. 14

FIG. 11–14.—Adrenal cortex of a healthy 5-day old rat. The extracellular apoptotic body illustrated in Fig. 11 shows closely aggregated but apparently intact mitochondria of epithelial cell type (M) and several lipid globules (L). Dense granular nuclear fragments (N) in extracellular bodies are depicted in Fig. 12. A well-preserved apoptotic body within a " histiocyte " (H) is shown in Fig. 13: the long arrow points to the membrane of the body and the short arrow to the phagosome membrane. Fig. 14 illustrates a partly degraded apoptotic body in a " histiocyte ", which lies between an epithelial cell (E) and collagen fibres (C). Fig. 11: ×17,000; Fig. 12: ×14,000; Fig. 13: ×13,000; Fig. 14: ×10,500.

Fig. 15

Fig. 16

Fig. 17

Fig. 18

FIG. 15–18.—Paracordal mesoderm of a rat embryo killed 24 hours after administration of the teratogen 7-hydroxymethyl-12-methylbenz(a)-anthracene to the mother on day 13 of pregnancy. Two extracellular apoptotic bodies are shown in Fig. 15: note their compact cytoplasm and contrast the density of their nuclear remnants with the loose texture of the chromatin in the nucleus (N) of the adjacent intact mesenchymal cell. Fig. 16 and 17 show apoptotic bodies within the cytoplasm of mesencyhmal cells: their ribosomes are closely aggregated and mitochondria (M) display autolytic changes. A large complex lysosomal residual body, which is the result of fusion of a number of lysosomes containing remnants of phagocytosed apoptotic bodies in a mesenchymal cell, is illustrated in Fig. 18. Fig. 15: ×9200; Fig. 16: ×18,000; Fig. 17: ×23,500; Fig. 18: ×9500.

FIG. 19.—Adrenal cortex of a rat foetus killed after thrice-daily administration of 1 mg prednisolene to the mother for 3 days, starting on the 17th day of gestation. An apoptotic body derived from an epithelial cell lies within the cytoplasm of a sinusoid-lining cell. Its mitochondria show early autolytic changes. $\times 16,000$. Inset: $\times 25,500$.

Fig. 20

Fig. 21

Fig. 22

FIG. 20–22.—Regressing Huggins rat mammary tumour examined 2 days after oophorectomy. Fig. 20 shows a well-preserved apoptotic body with nuclear remnants within the cytoplasm of an intact tumour cell; the phagocytosed body illustrated in Fig. 21 displays autolytic mitochondrial (M) changes: further degradation of ingested bodies is evident in Fig. 22. Fig. 20 and 21: × 16,000; Fig. 22: × 12,000.

take up large structures such as apoptotic bodies under physiological conditions.

Subsequent to their ingestion by other cells—whether these be embryonic or adult, " histiocytic " or epithelial, normal or neoplastic—apoptotic bodies undergo a process within phagosomes (Fig. 8 and 10; Kerr, 1971, 1972*a*; Currie *et al.*, 1972; Wyllie *et al.*, 1972*a*) that is ultrastructurally very similar to ischaemic coagulative necrosis (Kerr, 1970) and *in vitro* autolysis (Trump *et al.*, 1965) of whole cells. The matrix of mitochondria becomes electron-lucent and displays focal flocculent densities, the membranes of organelles and those bounding the bodies themselves break down and ribosomes become swollen and indistinct. It should be noted that this is the first stage at which the bodies exhibit changes that indubitably indicate cessation of co-ordinated metabolic activity (Trump and Ginn, 1969). The evidence suggests that lysosomes are not involved in the *genesis* of this degeneration (Fig. 8; Kerr, 1971, 1972*a*), and it seems likely that autolysis of phagocytosed apoptotic bodies is a result of their inability to maintain chemical homoeostasis within phagosomes: whether they are capable of prolonged " survival " in the extracellular space is unknown, since they are probably always either rapidly ingested or "sloughed off " from mucosal surfaces soon after their formation. Lysosomal enzymes do, however, play a vital role in the further degradation of phagocytosed bodies (Ballard and Holt, 1968; Kerr, 1971; Kerr and Searle, 1972*b*), and these are rapidly reduced to electron-dense lysosomal residual bodies (Fig. 9; Klion and Schaffner, 1966; Saunders, 1966; Saunders and Fallon, 1966; Farbman, 1968; Kerr, 1971; Currie *et al.*, 1972; Kerr and Searle, 1972*a* and *b*; Wyllie *et al.*, 1972*a*). The acquisition of lysosomal hydrolases by the phagosomes is associated with depletion of pre-existing secondary lysosomes in the cytoplasm of the ingesting cells (Fig. 9; Kerr, 1971; Kerr and Searle, 1972*b*), indicating that they fuse with the phagosomes. But there is little doubt that the presence of these phagosomes also evokes new synthesis of hydrolases (Ballard and Holt, 1968), and an increase in hydrolase content in response to the ingestion of apoptotic bodies has also been reported in neoplastic epithelial cells (Kerr and Searle, 1972*b*).

It is difficult to determine precisely the time taken for the sequence of events described above, since apoptosis is going on continuously in normal foetal and adult tissues and growing neoplasms; even when augmented by various stimuli, the process appears to start in individual cells of the same organ or tissue at different times. However, examination of the serial changes that take place in several experimental models (Kerr, 1971; Crawford *et al.*, 1972; Wyllie *et al.*, 1972*b*) suggests that the process is completed fairly rapidly: bodies may form and disappear within 24 hours. Partly degraded remnants of apoptotic bodies are difficult to discern histologically and electron microscopy shows that bodies that can be detected with the light microscope comprise only a small fraction of the total number of cell remnants present (Kerr and Searle, 1972*b*).

It is important to appreciate that the finding of relatively few apoptotic bodies in histological sections of a tissue means that quite extensive cell " drop-out " is taking place: mitotic figures have an analagous significance for cell proliferation.

Apoptosis is well suited to a role in tissue homoeostasis, since it can result in extensive deletion of cells with little tissue disruption. Following fragmentation of an affected cell, the remains are rapidly disposed of by nearby intact cells. There is no inflammation, as is elicited by coagulative necrosis, and even the lysosomal residual bodies soon disappear, possibly as a result of cell defaecation (Kerr, 1971, 1972*a*). Moreover, the process is economical in terms of re-utilization of cell components.

Fig. 23

Fig. 24

FIG. 23 and 24.—Untreated squamous cell carcinoma of the human cervix uteri. Apoptotic bodies with nuclear remnants are seen within carcinoma cells, which can readily be identified by the presence of tonofibrils (T). Autolytic changes are evident in the body illustrated in Fig. 23: more advanced degradation is shown in Fig. 24. Fig. 23: $\times$14,200; Fig. 24: $\times$16,000.

### THE OCCURRENCE AND IMPLICATIONS OF APOPTOSIS

The size of a cell population, whether neoplastic or not, depends on the balance between cell production and cell loss. An enormous amount of work has been done over many years on multiplication of cells under various circumstances; by contrast, relatively little attention has been paid to controlled cell deletion. We believe that enough is now known about the occurrence of apoptosis to establish it as an important, and possibly the only mode of controlled cell death, which contributes to the regulation of cell populations in a variety of mammalian tissues under many different conditions.

Small numbers of apoptotic bodies can be found in histological sections of many *healthy tissues* (Fig. 1 and 3*a*), and there seems little doubt that apoptosis plays an important role in the regulation of normal cell populations. The sparseness of the bodies in most tissues makes ultrastructural studies very difficult, but electron microscopy of the normal neonatal rat adrenal cortex, where apoptosis is enhanced as a result of temporary physiological ACTH-deprivation (Wyllie *et al.*, 1972*a*), shows the process to conform exactly (Fig. 11–14) to the general description already given. Much more work is needed to determine the extent and frequency of apoptosis in the organs and tissues of healthy adult animals, and practically nothing is known about the factors that determine which cells will be affected.

An interesting though poorly understood manifestation of apoptosis in healthy animals is the occurrence of so-called tingible bodies in the germinal centres of lymphoid follicles. They show the typical histological features of apoptotic bodies, and examination of electron micrographs published by Swartzendruber and Congdon (1963) indicates to us that they undergo the classic sequence of changes following phagocytosis. Many of them have been shown to be derived from cells that have recently synthesized DNA (Fliedner, 1967; Odartchenko *et al.*, 1967) and it has been suggested that cell death in lymphoid follicles might be an inevitable consequence of rapid cell proliferation (Yoffey and Courtice, 1970). However, we have not been able to detect an increase in apoptosis in the regenerating liver remnant examined at varying times after partial hepatectomy in the rat, and in the embryo the distribution of apoptotic cell deletion has been shown to be focal and highly specific, and is certainly not merely a manifestation of rapid cell multiplication (Glücksmann, 1951; Saunders, 1966; Menkes, Sandor and Ilies, 1970).

Perhaps because of its frequently massive dimensions in *the embryo*, the significance of apoptosis in vertebrate ontogeny was recognized early, and recent electron microscope studies (Fig. 15–18; Saunders and Fallon, 1966; Farbman, 1968; Webster and Gross, 1970; Crawford *et al.*, 1972) have shown that the ultrastructural features of the embryonic process are essentially the same as those observed during post-natal life. The morphological appearances have not, however, always been correctly interpreted: as recently as 1970, Menkes and his colleagues suggested that Feulgen-positive, basophilic " necrospherules " within the cytoplasm of other embryonic cells might represent signs of early degeneration of the latter, whereas their structure seems to us typical of phagocytosed apoptotic bodies. A similar misconception may to some extent account for the delay in the appreciation of the significance of apoptosis in adult tissues and neoplasms: as will be emphasized later, it is likely that apoptotic bodies within heterophagosomes have sometimes been mistakenly identified as autophagic vacuoles.

Focal apoptosis plays a vital role in many normal embryonic processes such as the development of the lumina of tubular structures, the fashioning of limbs, the formation of interdigital clefts, and the involution of phylogenetic vestiges (Glücksmann, 1951; Saunders, 1966;

Menkes *et al.*, 1970). Its appearance is precisely controlled, probably by diffusible substances: the susceptibility of groups of cells depends on their position in the embryo and the stage of development that has been reached (Saunders, 1966; Saunders and Fallon, 1966). It is interesting that hormones that cause proliferation and differentiation of cells in some situations may cause, at the same time, apoptosis of others: for example, in developing amphibians, thyroid hormone stimulates both general body growth and widespread cell death in the gills and tail (Saunders, 1966).

Apart from its role in normal ontogenesis, apoptosis is also important in *teratogenesis*. A number of teratogenic agents have been found to produce massive apoptosis at their site of action (Fig. 15–18; Menkes *et al.*, 1970; Crawford *et al.*, 1972), and in at least some cases, the subsequent congenital malformations have been fully accounted for by the distribution of focal apoptosis observed within a few hours of treatment (Menkes *et al.*, 1970; Crawford *et al.*, 1972).

In the pathogenesis of such congenital malformations, apoptosis greatly exceeds mitosis in localized areas, effecting a net reduction in cell numbers. By contrast, in *growing malignant neoplasms* cell numbers progressively increase. Nevertheless, as has already been pointed out, many recent studies have shown that spontaneous and continuous death of cells is an inherent property of malignant neoplasms. However, there has been little detailed investigation of the associated morphology; the foci of coagulative necrosis seen in some lesions obviously could not account for the kinetic data.

Apoptotic bodies have been found histologically in all the malignant neoplasms we have examined (Fig. 4) and they are frequently extremely numerous. Many are rather inconspicuous and some practice is needed to identify the smaller bodies with the light microscope, especially those without a nuclear component.

Electron microscope studies, so far performed on human basal (Kerr and Searle, 1972*a* and *b*) and squamous (Searle, unpublished observations) cell carcinomata, and on Huggins rat mammary tumours induced by 7,12-dimethylbenz(a)anthracene (DMBA) (Currie *et al.*, 1972), have shown that the apoptotic bodies are essentially the same in structure as those derived from non-neoplastic cells and that whilst a few of the bodies are taken up by "histiocytes", the majority are rapidly phagocytosed by intact tumour cells (Fig. 20 and 23). Progressive stages in the degradation of the bodies by tumour cells can be traced with the electron microscope (Fig. 21, 22 and 24) and the participation of lysosomal hydrolases synthesized in the tumour cells has been substantiated in the case of basal cell carcinomata by the application of electron histochemical techniques (Kerr and Searle, 1972*b*).

The kinetic significance of finding moderate numbers of apoptotic bodies in histological sections of a tissue has already been stressed, and electron microscopy of basal cell carcinomata leaves no doubt that apoptosis accounts for extensive and continuous deletion of cells from these tumours (Kerr and Searle, 1972*a* and *b*). Both apoptotic bodies and mitotic figures are sometimes numerous in rapidly growing tumours; it is the balance between the two processes that determines the rate of enlargement.

The spontaneous occurrence of apoptosis in growing malignant neoplasms suggests that it might also be implicated in some types of therapeutically induced *tumour regression*, but few investigations have so far been undertaken. Preliminary studies of human squamous cell carcinomata indicate that there is an increase in apoptosis after irradiation (Kerr and Searle, 1972*b*), and the regression of Huggins rat mammary tumours that follows oophorectomy has been shown to be associated with extensive and diffuse apoptotic deletion of tumour cells (Currie *et al.*, 1972). A decrease in the size of individual cells is, of course, also often of great importance (Scott, Christian and Currie, 1967) and autophagocytosis probably plays a part in its genesis (Scott *et al.*, 1967; Anton and Brandes, 1968; Paris and Brandes, 1971). However, it seems to us likely that some of the structures that have been identified as autophagic vacuoles in the past might really have been ingested apoptotic bodies. It is possible that histological assessment of the " apoptotic index " of a tumour several days after the commencement of therapy might, in some cases, provide a useful measure of its response, but more experimental work is needed before this suggestion is applied to man for the situation is complex and cells may also be deleted in other ways (Scott *et al.*, 1967).

A rather similar situation pertains in the *atrophy and involution* of non-neoplastic tissues and organs, where a decrease in cell numbers is probably often as important as a decrease in the size of individual cells. Whilst it has been suggested that autophagocytosis might contribute to the development of the latter (Helminen, Ericsson and Niemi, 1970; Cole, Matter and Karnovsky, 1971; Helminen and Ericsson, 1971), little or no attention has been paid to the way in which cells are deleted.

We have found that apoptosis plays a significant part in at least some types of atrophy and involution. Perhaps the most thoroughly studied is the gross and rapid reduction in size of rat liver lobes that follows ligation of their portal blood supply; here there is enhancement of autophagocytosis associated with diffuse shrinkage of hepatocytes within hours of operation (Cole *et al.*, 1971), and this is followed by massive apoptotic deletion of liver cells, which reaches a peak several days later (Fig. 6–10; Kerr, 1971). Apoptosis is also prominent in certain differentiated tissues undergoing atrophy as a result of withdrawal or administration of hormones. Thus, following a reduction in the blood concentration of ACTH in rats, a shower of apoptotic bodies appears in the adrenal cortex (Fig. 19), an occurrence that can be prevented by administration of exogenous ACTH (Wyllie *et al.*, 1972*b*); several days after orchidectomy in the rat, numerous apoptotic bodies are seen amongst the somewhat shrunken epithelial cells that line the prostatic glandular acini (Fig. 2); published descriptions of the process responsible for the deletion of lymphoid cells that follows large doses of glucocorticoids (Haelst, 1967; Makman, Nakagawa and White, 1967; Abraham, Morris and Hendy, 1969; La Pushin and de Harven, 1971) indicate to us that this is also an example of apoptosis. Finally, apoptotic bodies are quite numerous in the involuting human corpus luteum (Searle, unpublished observations).

There is little doubt that phagocytosed apoptotic bodies have sometimes been mistaken for autophagic vacuoles in electron microscopic studies of atrophy and involution (see, for example, Fig. 6 in Helminen and Ericsson, 1971). The distinction may be difficult unless recognizable nuclear remnants are present in the ingested apoptotic bodies (Kerr, 1972*a*), but it is crucial to the correct understanding of many problems in cell population kinetics.

The evidence for the participation of apoptosis in the various types of cell population change considered so far has been founded on detailed observation. We should now like to speculate that *hyperplasia* might sometimes result from decreased apoptosis rather than increased mitosis, although we emphasize that we know of no definitive studies that support such an hypothesis. It seems to us possible that focal hyperplasia in tissues subject to cyclic hormonal stimulation such as the breast might be due to failure of clones of cells to respond in the normal way to falling hormone concentrations by undergoing apoptosis.

We have an impression that apoptotic bodies are rare in benign neoplasms, but much more work is required to show whether this is a constant finding.

In view of what has been said about the occurrence of apoptosis under physiological conditions, it is perhaps somewhat surprising to find that it is also augmented in "*tissue injury*", often developing in association with focal coagulative necrosis, both *in vivo* (Kerr, 1971) and *in vitro* (L. T. Hou, personal communication). Electron microscopic studies of the augmented apoptosis occurring in a variety of types of liver injury (Biava and Mukhlova-Montiel, 1965; Klion and Schaffner, 1966; Moppert, Ekesparre and Bianchi, 1967; Kerr, 1969, 1970, 1971) show that the morphological changes conform to the usual stereotyped pattern, and ultrastructurally typical apoptosis can be induced in the rat adrenal cortex by administration of the adrenocorticolytic agent DMBA (Kerr, 1972*b*). Examination of published electron micrographs of so-called individual-cell dyskeratosis produced in the epidermis by ultraviolet irradiation (Wilgram *et al.*, 1970) suggests to us that this is also an example of apoptosis.

It is evident that certain agents (hepatotoxins, electromagnetic radiation, DMBA) are capable of inducing either coagulative necrosis or apoptosis of cells, as well as having both carcinogenic and teratogenic properties. However, the significance of this observation is obscure.

### FACTORS INITIATING APOPTOSIS

Little is known of the factors that initiate apoptosis or of the nature of the cellular mechanisms activated before the appearance of the characteristic morphological changes. It seems clear, however, that in certain circumstances apoptosis is an inherently programmed event, determined by *intrinsic* "*clocks*" specific for the cell type involved. Thus in avian embryonic tissue explants apoptosis occurred in susceptible zones "on schedule" (Saunders and Fallon, 1966). But even here, some degree of environmental control is evident; diffusible substances from adjacent tissues are capable of delaying the process, sometimes indefinitely (Saunders and Fallon, 1966).

Although the nature of many of these diffusible substances is still obscure, steroid *hormones* are known to affect apoptosis in the Müllerian ducts of chick embryos, oestrogenic steroids inhibiting the expected Müllerian regression in genetic males, and androgenic steroids promoting regression in genetic females (Menkes *et al.*, 1970). As we have already shown, the role of hormones in modulating apoptosis is not restricted to ontogenesis; apoptosis can be promoted or inhibited in certain differentiated mammalian tissues by hormone withdrawal or stimulation.

The mode of action of triggers and inhibitors of apoptosis is unknown, but it is tempting to speculate that it might involve stimulation of messenger RNA and protein synthesis. If indeed apoptosis depends on expression of part of the genome, which is normally repressed in viable cells, the initiation of such a stereotyped series of changes by a wide variety of stimuli would be understandable.

It is obvious that much work has still to be done on the factors that determine the occurrence and extent of apoptosis in malignant neoplasms. Ischaemia probably accounts for the dense clusters of apoptotic bodies often seen in the neighbourhood of coagulative necrosis. However, we know little about the aetiology of diffuse apoptosis: nutritional, hormonal and immune factors, and intrinsic controls, perhaps including cell ageing, may be involved.

### THE CONCEPT OF APOPTOSIS: CONCLUSIONS

Though certain of the morphological manifestations of apoptosis have long been recognized at the light microscope level, its ultrastructural features have been described only recently. The implications of the process in tissue kinetics have not hitherto been appreciated, except by embryologists. We believe that the evidence presented here establishes it not only as a distinctive morphological process but also as an important basic biological phenomenon, which plays a complementary but opposite role to mitosis in the regulation of animal cell populations. Thus, it is involved in cell turnover in many normal tissues and accounts for extensive spontaneous cell loss in malignant neoplasms; it is implicated in tissue atrophy and involution and it plays a part in at least some types of tumour regression; it is of major importance in both normal ontogenesis and teratogenesis. The ultrastructural features of apoptosis and its initiation and inhibition by a variety of environmental stimuli suggest to us that it is an active, controlled process.

Many challenging problems obviously remain to be solved, the factors that determine which cells will be affected, the role of ageing, the mode of action of initiators and inhibitors, the earliest biochemical and morphological events, and the mechanism of the cellular condensation being but a few.

This work was supported in part by the Scottish Hospital Endowments Research Trust and in by part the National Health and Medical Research Council of Australia. A. H. W. held a Junior Research Fellowship of the Medical Research Council. We are grateful to Dr J. Searle and Mr D. Collins for providing Fig. 1, 23 and 24, and to Dr D. N. Wheatley for the histological sections of regenerating rat liver. We thank the following for their technical assistance: Mrs Marget Inglis, Mr Peter MacLennan, Mr George Milne, Mr Alastair McKinnon, Mr Robert Cardno, Miss Lynda Hardy and Mr Brian Harmon.

### REFERENCES

Abraham, R., Morris, M. & Hendy, R. (1969) Lysosomal Changes in Epithelial Cells of the Mouse Thymus after Hydrocortisone Treatment. *Histochemie*, **17**, 295.

Anton, E. & Brandes, D. (1968) Lysomes in Mice Mammary Tumors Treated with Cyclophosphamide. *Cancer, N.Y.*, **21**, 483.

Ballard, K. J. & Holt, S. J. (1968) Cytological and Cytochemical Studies on Cell Death and Digestion in the Foetal Rat Foot: the Role of Macrophages and Hydrolytic Enzymes. *J. Cell Sci.*, **3**, 245.

Biava, C. & Mukhlova-Montiel, M. (1965) Electron Microscopic Observations on Councilman-like Acidophilic Bodies and Other Forms of Acidophilic Changes in Human Liver Cells. *Am. J. Path.*, **46**, 775.

Clifton, K. H. & Yatvin, M. B. (1970) Cell Population Growth and Cell Loss in the MTG-B Mouse Mammary Carcinoma. *Cancer Res.*, **30**, 658.

Cole, S., Matter, A. & Karnovsky, M. J. (1971) Autophagic Vacuoles in Experimental Atrophy. *Exp. mol. Pathol.*, **14**, 158.

Crawford, A. M., Kerr, J. F. R. & Currie, A. R. (1972) The Relationship of Acute Mesodermal Cell Death to the Teratogenic Effects of 7-OHM-12-MBA in the Fetal Rat. *Br. J. Cancer.* In press.

Currie, A. R., Kerr, J. F. R., Scott, G. B. & Inglis, M. S. (1972) Regression of Endocrine-dependent Mammary Tumours: the Role of Apoptosis. *Br. J. Cancer.* In press.

Farbman, A. I. (1968) Electron Microscope Study of Palate Fusion in Mouse Embryos. *Devl Biol.*, **18**, 93.

Fliedner, T. M. (1967) On the Origin of Tingible Bodies in Germinal Centers. In *Germinal Centers in Immune Responses.* Ed. H. Cottier, N. Odartchenko, R. Schindler and C. C. Congdon. Berlin: Springer. p. 218.

Frindel, E., Malaise, E. & Tubiana, M. (1968) Cell Proliferation Kinetics in Five Human Solid Tumors. *Cancer, N.Y.*, **22**, 611.

Glücksmann, A. (1951) Cell Deaths in Normal Vertebrate Ontogeny. *Biol. Rev.*, **26**, 59.

Goldsmith, M. (1966) The Anatomy of Cell Death. *J. Cell Biol.*, **31**, 41A.

Haelst, U. van (1967) Light and Electron Microscopic Study of the Normal and Pathological Thymus of the Rat. II The Acute Thymic Involution. *Z. Zellforsch. mikrosk. Anat.*, **80**, 153.

Helminen, H. J. & Ericsson, J. L. E. (1971) Ultrastructural Studies on Prostatic Involution in the Rat. Mechanism of Autophagy in Epithelial Cells, with Special Reference to the Rough-surfaced Endoplasmic Reticulum. *J. Ultrastruct. Res.*, **36**, 708.

Helminen, H. J., Ericsson, J. L. E. & Niemi, M. (1970) Lysosomal Changes during Castration—induced Prostatic Involution in the Rat. *Acta path. microbiol. scand.*, **78A**, 493.

Iversen, O. H. (1967) Kinetics of Cellular Proliferation and Cell Loss in Human Carcinomas. A Discussion of Methods Available for *in vivo* Studies. *Eur. J. Cancer*, **3**, 389.

Judah, J. D., Ahmed, K. & McLean, A. E. M. (1965) Pathogenesis of Cell Necrosis. *Fedn Proc. Fedn Am. Socs exp. Biol.*, **24**, 1217.

Kerr, J. F. R. (1965) A Histochemical Study of Hypertrophy and Ischaemic Injury of Rat Liver with Special Reference to Changes in Lysosomes. *J. Path. Bact.*, **90**, 419.

Kerr, J. F. R. (1967) Lysosome Changes in Acute Liver Injury due to Heliotrine. *J. Path. Bact.*, **93**, 167.

Kerr, J. F. R. (1969) An Electron-microscope Study of Liver Cell Necrosis due to Heliotrine. *J. Path.*, **97**, 557.

Kerr, J. F. R. (1970) An Electron Microscopic

Study of Liver Cell Necrosis due to Albitocin. *Pathology*, **2**, 251.

KERR, J. F. R. (1971) Shrinkage Necrosis: a Distinct Mode of Cellular Death. *J. Path.*, **105**, 13.

KERR, J. F. R. (1972*a*) Some Lysosome Functions in Liver Cells Reacting to Sublethal Injury. In *Lysosomes in Biology and Pathology*, Vol. 3. Ed. J. T. Dingle and Honor B. Fell. Amsterdam: North-Holland. In press.

KERR, J. F. R. (1972*b*) Shrinkage Necrosis of Adrenal Cortical Cells. *J. Path.* In press.

KERR, J. F. R. & SEARLE, J. (1972*a*) A Suggested Explanation for the Paradoxically Slow Growth Rate of Basal-cell Carcinomas that Contain Numerous Mitotic Figures. *J. Path.* In press.

KERR, J. F. R. & SEARLE, J. (1972*b*) The Digestion of Cellular Fragments within Phago-lysosomes in Carcinoma Cells. *J. Path.* In press.

KLION, F. M. & SCHAFFNER, F. (1966) The Ultrastructure of Acidophilic "Councilman-like" Bodies in the Liver. *Am. J. Path.*, **48**, 755.

LAIRD, A. K. (1969) Dynamics of Growth in Tumors and in Normal Organisms. In *Human Tumor Cell Kinetics*. Ed. S. Perry. Natn. Cancer Inst. Monogr., No. 30. p. 15.

LALA, P. K. (1971) Studies on Tumor Cell Population Kinetics. In *Methods in Cancer Research*, Vol. 6. Ed. H. Busch. New York: Academic Press. p. 3.

LALA, P. K. (1972) Evaluation of the Mode of Cell Death in Ehrlich Ascites Tumor. *Cancer, N.Y.*, **29**, 261.

LA PUSHIN, R. W. & DE HARVEN, E. (1971) A Study of Gluco-corticosteroid-induced Pyknosis in the Thymus and Lymph Node of the Adrenalectomized Rat. *J. Cell Biol.*, **50**, 583.

MAKMAN, M. H., NAKAGAWA, S. & WHITE, A. (1967) Studies of the Mode of Action of Adrenal Steroids on Lymphocytes. *Recent Prog. Horm. Res.*, **23**, 195.

MENKES, B., SANDOR, S. & ILIES, A. (1970) Cell Death in Teratogenesis. In *Advances In Teratology*, Vol. 4. Ed. D. H. M. Woollam. London: Logos Press. p. 169.

MOPPERT, J., EKESPARRE, D. V. & BIANCHI, L. (1967) Zur Morphogenese der eosinophilen Einzelzellnekros im Leberparenchym des Menschen. Eine licht-und elektronenoptisch korrelierte Untersuchung. *Virchows Arch. path. Anat. Physiol.*, **342**, 210.

ODARTCHENKO, N., LEWERENZ, M., SORDAT, B., ROOS, B. & COTTIER, H. (1967) Kinetics of Cellular Death in Germinal Centers of Mouse Spleen. In *Germinal Centers in Immune Responses*. Ed. H. Cottier, N. Odartchenko, R. Schindler and C. C. Congdon. Berlin: Springer. p. 212.

PARIS, J. E. & BRANDES, D. (1971) Effect of X-irradiation on the Functional Status of Lysosomal Enzymes of Mouse Mammary Gland Carcinomas. *Cancer Res.*, **31**, 392.

REFSUM, S. B. & BERDAL, P. (1967) Cell Loss in Malignant Tumours in Man. *Eur. J. Cancer*, **3**, 235.

SAUNDERS, J. W. (1966) Death in Embryonic Systems. *Science, N.Y.*, **154**, 604.

SAUNDERS, J. W. & FALLON, J. F. (1966) Cell Death in Morphogenesis. In *Major Problems in Developmental Biology*. Ed. M. Locke. New York: Academic Press. p. 289.

SCOTT, G. B., CHRISTIAN, H. J. & CURRIE, A. R. (1967) The Huggins Rat Mammary Tumors: Cellular Changes Associated with Regression. In *Endogenous Factors Influencing Host–Tumor Balance*. Ed. R. W. Wissler, T. L. Dao and S. Wood, Jr. Chicago: University of Chicago Press. p. 99.

STEEL, G. G. (1967) Cell Loss as a Factor in the Growth Rate of Human Tumours. *Eur. J. Cancer*, **3**, 381.

SWARTZENDRUBER, D. C. & CONGDON, C. C. (1963) Electron Microscope Observations on Tingible Body Macrophages in Mouse Spleen. *J. Cell Biol.*, **19**, 641.

TRUMP, B. F. & GINN, F. L. (1969) The Pathogenesis of Subcellular Reaction to Lethal Injury. In *Methods and Achievements in Experimental Pathology*, Vol. 4. Ed. E. Bajusz and G. Jasmin. Basel: Karger, p. 1.

TRUMP, B. F., GOLDBLATT, P. J. & STOWELL, R. E. (1965) Studies on Necrosis of Mouse Liver *in vitro*. Ultrastructural Alterations in the Mitochondria of Hepatic Parenchymal Cells. *Lab. Invest.*, **14**, 343.

WEBSTER, D. A. & GROSS, J. (1970) Studies on Possible Mechanisms of Programmed Cell Death in the Chick Embryo. *Devl Biol.*, **22**, 157.

WEINSTEIN, G. D. & FROST, P. (1970) Cell Proliferation in Human Basal Cell Carcinoma. *Cancer Res.*, **30**, 724.

WILGRAM, G. F., KIDD, R. L., KRAWCZYK, W. S. & COLE, P. L. (1970) Sunburn Effect on Keratinosomes. A Report with Special Note of Ultraviolet-induced Dyskeratosis. *Archs Derm.*, **101**, 505.

WYLLIE, A. H., KERR, J. F. R. & CURRIE, A. R. (1972*a*) Cell Death in the Normal Neonatal Rat Adrenal Cortex. *J. Path.* (To be published).

WYLLIE, A. H., KERR, J. F. R., MACASKILL, I. A. M. & CURRIE, A. R. (1972*b*) Adrenocortical Cell Deletion: the Role of ACTH. *J. Path.* (To be published).

YOFFEY, J. M. & COURTICE, F. C. (1970) *Lymphatics, Lymph and the Lymphomyeloid Complex*. New York: Academic Press. p. 534.